# Returning to Work After Sick Leave – The Role of Work Demands and Resources, Self-Efficacy, and Social Support

**DOI:** 10.3389/fpsyg.2020.00661

**Published:** 2020-04-16

**Authors:** Eva Boštjančič, Kaja Galič

**Affiliations:** Department of Psychology, Faculty of Arts, University of Ljubljana, Ljubljana, Slovenia

**Keywords:** return to work, sick leave, social engagement, self-efficacy, job demands and resources, perceived control, social support

## Abstract

Returning to work after sick leave is a process that begins with the initial steps of functional recovery and results in full vocational capacity. Different personal and situational factors could influence an employee’s well-being after returning. The following research was conducted in order to examine how occupational demands and resources, self-efficacy, and social engagement contribute to the return-to-work process. A total of 256 employees took part in the study, who were later divided into two groups: short- (less than 30 days), and long-term (more than 30 days) sick leave. We measured their self-efficacy at the workplace, recent job demands and resources, social engagement, and work satisfaction after returning to work after sick leave. The results showed that personal (gender, age, and self-efficacy), social (social engagement), and occupational (job demands and resources) factors are associated with the duration of sick leave. Participants who were on shorter sick leaves reported being more satisfied with their work after returning than those returning from long-term sick leave. The research presents important insights that could help employers better understand the needs of employees who are returning to work after sick leave.

## Introduction and Objectives

The majority of people consider the workplace a crucial source of social capital, support, and life purpose (Rydström et al., 2017). Moreover, employment is often seen as one of the most important predictors of quality of life (Passier et al., 2011). Employment contributes to adult identities, confers financial benefits and status, and in general improves the quality of life and reduces ill health (National Clinical Guideline Centre (UK), 2013). Returning to work after sick leave does not necessarily mean that someone has fully recovered from their health concerns (De Rijk et al., 2009). Although the term “returning to work” is commonly used, there are many different definitions. Young et al. (2005) describe it as a complicated and evolving process, which can be viewed and described from many different perspectives. They offer two explanations for this. On the one hand, returning to work can be viewed as an outcome, e.g., *de facto* starting to work again; on the other hand, it can also mean a process, which begins with the initials steps of functional recovery and results in full vocational capacity (Young et al., 2005). For this research, the first definition was used.

The aim of this study is to look more broadly, across various environment’s factors connected with returning to work after an absence due to sickness. Further, the goal is to provide information for employers and help them better understand and support their employees in this challenging transition period. In other words, to point out which are the factors that could help to buffer the negative effects of sick leave.

Based on a systematic survey of the literature on returning to work, we concluded that there are some factors that influence the process significantly more than others: general self-efficacy, social support, job demands, and perceived control. Therefore, we will now briefly describe the factors mentioned above.

### Return to Work and Self-Efficacy

Self-efficacy plays an important role in facilitating the process of returning to work (Volker et al., 2015; Lork and Holmgren, 2018). It can be described as an optimistic sense of one’s personal competences, and there is a significant correlation between self-efficacy, motivation, and accomplishments (Scholz et al., 2002). Amick et al. (2004) found that increased self-efficacy together with perceived support from the workplace were among the most important factors for returning to work after a 6-month sick leave. In a different study, Katz et al. (2005) discovered that self-efficacy played an important role if the period of sick leave was between 2 and 6 months, but, if the absence was longer than 1 year, a supportive workplace had a more positive affect than self-efficacy. Busch et al. (2007) pointed out that low self-efficacy can more than double the chance that an employee will go on sick leave again. Dionne et al. (2007) found out that higher self-efficacy acted as a protective factor if an employee’s attempt to return to work was unsuccessful.

Lork and Holmgren (2018) conducted a study focusing on a specific construct, return-to-work self-efficacy. They described this as the belief that one has the capacity to meet the demands that are required to return to work. They found that participants’ experience with health issues and therefore being on sick leave shaped their return-to-work self-efficacy. The absence of health and being on sick leave resulted in lower return-to-work self-efficacy. On the other hand, gaining back good health resulted in increased return-to-work self-efficacy.

Etuknwa et al. (2019) conducted a systematic review in order to evaluate the impact of key personal as well as social factors that contribute to sustainable return to work among individuals with musculoskeletal disorders and common mental disorders. They concluded that sustainable return to work was associated with self-efficacy. Out of seven studies, which focused on examining the effects of self-efficacy and the returning to work process, all of them provided moderate evidence suggesting that those employees with higher self-efficacy during their return to work have a higher likelihood of sustainable return to work than those with lower perceived self-efficacy (Etuknwa et al., 2019).

### Return to Work and Social Support

Having social support from one’s family can help in coping with health concerns. Furthermore, cooperation between various stakeholders in the return to work process – employees, employer, health professionals, etc., – has been proven to have a positive effect on the whole process (Dionne et al., 2007; Hoefsmit et al., 2014). Hoefsmit et al. (2014) found social support from relatives to be one of the environmental factors affecting the process, wherefore it, i.e., social support, can be beneficial and can aid the process of returning to work. Their research also showed that a lack of cooperation between employees, HR professionals, and supervisors, curative professionals and others, can also result in the prolongation of sick leave (Hoefsmit et al., 2014). Social support is also vital for those employees returning to work after suffering from burnout syndrome. Boštjančič and Koračin (2014) found that employees could receive the necessary support from their family and co-workers, especially in terms of understanding them and partially adapting their responsibilities at work when they return. Overall, they found out that family and co-workers can play an essential role in supporting individuals who are returning to work.

Lysaght and Larmour-Trode (2008) came to similar conclusions; they reported that moral support, assistance, general interest, and understanding are among the most important factors concerning whether employees on sick leave will return to work or not. Workers with supervisors and management that were unsympathetic to their needs appeared less motivated to take on difficult tasks when dealing with injuries; the opposite was true when they had managers who were cooperative. For those workers who were respected by their colleagues it seemed that the quality of that relationship contributed to a successful return to work. The participants also reported that having a proactive and involved supervisor, good communication, and cooperative climate were among the key factors that contributed to a successful return-to-work process. Lysaght and Larmour-Trode (2008) concluded that emotional support was one of the most important features that helped in returning to work.

Watt et al. (2015) investigated the relationship between social support and a sustainable return to work process among employees returning after occupational injury. Their sample included 110 participants who were asked about their return to work status, relationships with their managers and co-workers and social support outside the workplace. They concluded that social support at the workplace is among the most important interventions when it comes to ensuring the sustainable return to work process.

### Return to Work, Job Demands, and Perceived Control

The demand-control model is among the most widely used models in describing the impact that the psychosocial work environment has on employee health. Psychological job demands are defined as work pressure and workload. Decision control, on the other hand, is described as the range of skills required on the job and the social authority of each employee when it comes to decision-making (Haveraaen et al., 2016). Haveraaen et al. (2016) conducted a longitudinal study with 543 sick-listed employees. Their aim was to assess the association between psychological work characteristics and how fast the individuals returned to work after completing the return-to-work program. Employees who reported high psychological job demands had lower return-to-work rates than those who reported low job demands. In other words, high psychological work demands were associated with a delay in returning to work. Moreover, employees who reported having high decision control had significantly higher return-to-work rates than those reporting low decision control. In conclusion, having high psychological job demands and low decision control were both important predictors of delayed returns to work (Haveraaen et al., 2016).

Our hypothesis is that there will be significant differences in the duration of sick leave associated with the following variables – work demands and resources, self-efficacy, and self-support. High job demands and low decision control will correlate significantly with longer sick leaves. In contrast, high perceived self-efficacy and social support will correlate significantly with shorter sick leaves. While referring to the latter we mean those participants who were on a sick leave for less than 30 days, and when referring to long-term sick leave we mean those who were absent from work for more than 30 days.

The research question was oriented toward seeking an answer as to which personal (gender, age, self-efficacy), social (social engagement), and work-related (job demands and resources) factors could influence higher work satisfaction after long-term sick leave.

## Materials and Methods

### Participants and Procedure

Participants were selected by using the snowball method. In total, the sample consisted of 256 employees working in the Republic of Slovenia (Table 1), and were on sick leave in the previous year (191 females and 65 males). The mean age was 41.4 years (*SD* = 9.56). The average duration of their sick leave was 184.7 days, with the median of 64 days.

**TABLE 1:** Participants’ characteristics (*N* = 256).

**Variables**	**Whole sample (*N* = 256)**	**Employees experiencing short sick leave (*n* = 110)**	**Employees experiencing long sick leave (*n* = 146)**
**Gender**			
Females	74.6%	58.2%	87.0%
Males	25.4%	41.8%	13.0%
Age	*M* = 41.4, *SD* = 9.56	*M* = 37.5, *SD* = 9.8	*M* = 44.2, *SD* = 8.5
**Family**			
Living alone	5%	6%	4%
Number of people in household	*M* = 3.4, *SD* = 1.2	*M* = 3.3, *SD* = 1.2	*M* = 3.5, SD = 1.2
Number of days on sick leave	*Me* = 64.0, *IQR* = 293.7	*Me* = 5.0, *IQR* = 7.0	*Me* = 232.0, *IQR* = 337.5
Number of days after returning back to work	*Me* = 150.0, *IQR* = 300.0	*M*e = 105.0, *IQR* = 330.0	*Me* = 180.0, *IQR* = 300.0

*M, mean; Me, median; SD, standard deviation; IQR, interquartile range.*

The majority of participants listed illness as the reason for their sick leave (*N* = 188; 72%). In more detail, infectious and parasitic diseases (*N* = 34), cancer (*N* = 26), musculoskeletal and connective tissues disorders (*N* = 19), and behavioral and mental disorders (*n* = 17) were the most reported causes. There were 8% (*N* = 21) on sick leave due to an injury that happened outside the workplace, and 3% (*N* = 9) reported were on sick leave to take care of a family member. Additionally, 14% (*N* = 37) were on sick leave for other reasons than those mentioned. In more detail, most of them listed the following reasons: maternity leave (*N* = 14), burnout (*N* = 9), and surgery (*N* = 4). In Slovenia, maternity leave generally begins 28 days before the estimated date of birth and lasts 105 days. After maternity leave, parental leave can last for a further 260 days (eUprava, 2018). Thus, a woman can be absent from work for 365 days. Due to a similar dynamic with sick leave, we also included this group in our study.

The data were collected via an online survey tool^1^. The participants completed an online questionnaire including measures of self-efficacy, job demands and resources, social engagement, and work satisfaction upon returning to work after sick leave. The data was collected from March 2018 until February 2019. The average time needed to complete the questionnaire was 7 min and 33 s. The research participants were selected on a voluntary basis. We invited various non-profit organizations in Slovenia supporting patients with a variety of conditions (e.g., breast cancer, burnout, etc.) to take part in our study. Moreover, we promoted our research on forums (e.g., a forum for pregnant women and new mothers) and on LinkedIn and Facebook groups created to support patients during their sickness. The response rate was 17%, calculated based on everyone who opened the link to the questionnaire.

### Methods and Analyses

In order to determine on which variables to focus, a systematic literature review was conducted. The present research was designed using the exploration perspective. For data collection, we used a mixture of quantitative and qualitative approaches – the questionnaire, which consisted of four independent scales, and three open-ended question.

#### Self-Efficacy

We used the General Self-Efficacy Scale (GSE; Schwarzer and Jerusalem, 1995) to measure the participants’ self-reported self-efficacy. The instrument was developed to assess the general sense of perceived self-efficacy, with the goal of predicting the ability to cope with daily life challenges and adaptation after experiencing various stressful life events (GSE; Schwarzer and Jerusalem, 1995). The internal reliability of GSE, based on Cronbach’s alpha (from the original paper), is between 0.76 and 0.90. GSE is correlated with positive affective states, optimism, and self-satisfaction. It negatively correlates with depression, stress, health complaints, burnout, and anxiety.

#### Job Demands and Resources

In line with the job demands-resources model (Bakker and Demerouti, 2017), the Job Demands and Resources Questionnaire (JDRQ; Tement et al., 2010) is based on the Perceived Work Demand Scale (PWD; Boyar et al., 2007) and the Job Content Questionnaire (JCQ; Karasek et al., 1998). JDRQ includes five different job characteristics measured using 19 items on a five-point Likert scale ranging from 1 (strongly disagree) to 5 (strongly agree). The Cronbach’s alpha of this instrument (Tement et al., 2010) is 0.63 for work variety (three items; My job requires learning new things), 0.89 for perceived workload (five items; I feel like I have a lot of job demands), 0.80 for autonomy and the degree of decision-making freedom at work (three items; My job allows me to make decisions on my own), 0.88 for co-worker support (four items; My co-workers are willing to listen to my problems), and 0.93 for supervisor support (four items; My superiors are willing to support me).

#### Social Engagement

We used the Lubben Social Network Scale (LSNS-6; Lubben, 1988) with six questions to evaluate the respondents’ social capital (e.g., With how many of your relatives/friends are you able to talk about your personal matters/to how many can you turn when you need help?) and the frequency of their social connections (e.g., With how many of your relatives/friends are you in contact at least once per month?). The participants chose their answers from 0 (*no one*) to 5 (*10 or more*). The total score was calculated by calculating the average of all six items – a higher score indicating more social support. The internal reliability of the scale was 0.83 (Lubben, 1988).

#### Work Satisfaction After Returning From Sick Leave

We used one item for measuring work satisfaction after returning from sick leave (i.e., “Overall, I am pleased with my work after returning from sick leave”). The participants’ responses were recorded on five-point Likert scale that ranged from 1 (*completely disagree*) to 5 (*completely agree*).

At the end of the questionnaire, we added three open questions concerning personal opinions about coming back to work from sick leave: (1) What does returning to work after a long period of sick leave mean to you? (2) What does being on sick leave mean to you? (3) Describe your experience of returning to work.

The data were analyzed in several steps. First, for each variable a composite score was computed by averaging the respective items. The Pearson correlation was used to examine the association between variables. Using independent *t*-tests we analyzed the differences between participants’ experiences with short (less than 30 days) and long (30 and more days) sick leaves. Finally, multiple linear regression was employed to test the relationships between personal, social, and work-related factors and the duration of the sick leave. The duration was logarithmized before the analysis due to the highly skewed distribution. Multicollinearity was tested by the variance inflation factor (VIF). The highest VIF was 1.96, and thus no threat to multicollinearity was found. All associations were tested at the significance level of α = 0.05.

## Results

The means, standard deviations, and correlations of the research are presented in Table 2.

**TABLE 2:** Descriptive statistics and intercorrelations between observed variables (*N* = 256).

	***Mean***	***SD***	**1**	**2**	**3**	**4**	**5**	**6**	**7**	**8**	**9**
1. Age	41.37	9.65									
2. Number of days on sick leave	184.71	270.70	0.199**								
3. Work variety	2.10	0.66	−0.195**	–0.010							
4. Autonomy	3.11	1.15	0.204**	0.096	−0.251**						
5. Co-workers’ workplace support	3.31	1.10	–0.067	−0.197**	−0.155*	0.210**					
6. Leaders’ workplace support	3.15	1.29	–0.013	−0.165**	−0.176**	0.45**	0.581**				
7. Perceived workload	4.37	0.74	0.098	–0.043	−0.361**	0.022	−0.127*	−0.252**			
8. Self-efficacy	3.84	0.79	–0.127	−0.336**	0.032	0.078	0.242**	0.172**	0.019		
9. Social support/engagement	3.75	0.90	–0.050	–0.078	0.022	0.03	0.173**	0.152*	–0.046	0.192**	
10. Work satisfaction after sick leave	3.19	1.12	0.114	−0.198**	−0.175**	0.306**	0.458**	0.551**	−0.172**	0.255**	0.198**

*p < 0.05, **p < 0.01.

Work satisfaction after returning to work was significantly and positively correlated with perceived work autonomy, support from the workspace, significant others (partners, family members, and friends), as well as with perceived self-efficacy. Moreover, negative correlations between work satisfaction after extended sick leave were found with work variety, perceived workload, and length of absence.

A great number of participants (*N* = 151) shared their experience with returning to work. Summarizing their responses, most of them had negative experiences when returning to work. In more detail, participants reported the following problems: they struggled to catch up with the pace of work (*N* = 51); they were faced with more work and needed to catch up with missed work (*N* = 24); they did not feel part of the team anymore (*N* = 22); they pointed out there are no clear guidelines on how to help employees who are returning after long sick leaves (N = 17); feeling pressured (*N* = 15); a lot of stress (*N* = 14); a lack of understanding and support from superiors and co-workers (*N* = 14); resistance from superiors and co-workers, because of shorter work hours (*N* = 11 a lack of trust (*N* = 10); a feeling that sick people are perceived as a problem (*N* = 9); they were faced with many changes at work (*N* = 8); uncertainty (*N* = 8); they were moved to lower positions (*N* = 7); a feeling of stigma (*N* = 6); gossiping (*N* = 5); some of them even reported that things were so bad that they changed their job (*N* = 5). On the other hand, some reported positive experiences: satisfaction (*N* = 28); support from supervisors (*N* = 21); co-workers were prepared to help (*N* = 17); feeling accepted (*N* = 13); happiness that they were able to return after battling illness or dealing with another medical condition (*N* = 12); candid conversations with colleagues (*N* = 12); and modification of work schedules and tasks (*N* = 9).

We split the participants into two groups (shorter and longer sick leave) according to the duration of their sick leave, then compared their self-evaluations (Table 3). The results showed that there was a significant difference in perceived co-worker workplace support. Participants in the short-term sick leave group reported having higher support from their workplace than those who were on a longer sick leave. The results also showed significant differences between the groups regarding self-efficacy, and the self-evaluations of participants who were on a short-term sick leave showed higher perceived self-efficacy. Moreover, participants who were on a short sick leave also reported being more satisfied with their work after returning to work. Those who were on a longer sick leave reported having more autonomy at work.

**TABLE 3:** Differences in the sample means (*N* = 256) of work demands and resources, self-efficacy, social support, and work satisfaction after returning from sick leave.

	**Shorter sick leave (*n* = 110)**	**Longer sick leave (*n* = 146)**	**Differences between groups**
		
	**Mean and**		
	**standard**		
	**deviation**		
Work variety	2.14 (0.69)	2.07 (0.64)	*t* = 0.79; *p* = 0.430
Autonomy	2.93 (1.20)	3.24 (1.10)	*t* = -2.17; *p* = 0.031
Co-workers’ workplace support	3.53 (0.92)	3.15 (1.20)	*t* = 2.78; *p* = 0.006
Leaders’ workplace support	3.32 (1.24)	3.03 (1.32)	*t* = 1.83; *p* = 0.068
Perceived workload	4.43 (0.67)	3.63 (0.85)	*t* = 1.12; *p* = 0.263
Self-efficacy	4.12 (0.59)	3.63 (0.85)	*t* = 5.50; *p* < 0.001
Social support/engagement	3.88 (0.90)	3.66 (0.90)	*t* = 1.91; *p* = 0.057
Work satisfaction after sick leave	3.41 (0.94)	3.03 (1.22)	*t* = 2.67; *p* = 0.008

When controlling for other factors included in the regression model (Table 4), gender, age autonomy, perceived workload, self-efficacy, and work satisfaction after sick leave are all associated with the duration of the sick leave. Most of the participants with shorter sick leave were men, and had greater self-efficacy and work satisfaction upon returning to work. Sick leave was longer for older employees and those with greater work autonomy.

**TABLE 4:** Factors associated with the duration of sick leave (the results of multiple linear regression).

	**Std. reg. coef.**	***p*-value**
Male gender	–0.25	< 0.001
Age	0.24	< 0.001
Work variety	–0.01	0.860
Autonomy	0.18	0.002
Co-workers’ workplace support	–0.05	0.445
Leaders’ workplace support	–0.02	0.804
Perceived workload	–0.14	0.017
Self-efficacy	–0.24	< 0.001
Social support/engagement	–0.01	0.919
Work satisfaction after sick leave	–0.19	0.006

The participants were also asked to answer two open-ended questions: *What does returning to work after long sick leave mean to you? What does being on sick leave mean to you?* In addition, at the end of the questionnaire they were also invited to share their experience of returning to work.

In answer to the first question the participants reported that returning to work had mostly positive associations: a feeling of being useful and accepted, being back among people, being included again (*N* = 31), getting back to a daily routine and responsibilities (*N* = 19), income and financial security (*N* = 17), feelings of satisfaction (*N* = 12), physical exertion (*N* = 9), being healthy and getting better (*N* = 13), winning the battle against disease (*N* = 7), a feeling of happiness in being able to do what you love again (*N* = 6), the meaning of life and personal fulfilment (*N* = 5), security (*N* = 4), and new opportunities, challenges (*N* = 6). They also mentioned some negative outcomes: stress due to a lack of support from their superiors and the lack of autonomy, and thus additional worries (*N* = 21); additional work and burden (*N* = 5); less time and more money (*N* = 1); the need to catch up for lost time (*N* = 2); tiredness, the feeling of being used (*N* = 2); and fear of losing their job, constant concern (*N* = 2).

In answer to the second question the participants stated that being on sick leave meant having a lower income (*N* = 36), being able to rest and focus on getting better (*N* = 32), feelings of sadness, fear, concern, and powerlessness (*N* = 15), isolation, having less social interaction (*N* = 12), a burden (*N* = 10), stress (*N* = 8), an obstacle or set-back in one’s career (*N* = 8), being behind, lots of unfinished work (*N* = 7), feeling guilty (*N* = 6), pressure, more work when returning (*N* = 5), and working during their absence (*N* = 4). Individual responses mentioned losing value as an employee, stigma, sickness, boredom, a feeling of missing out, a loss or a big change, exertion, dissatisfaction, and also having more time.

## Discussion

Returning to work after sick leave is associated with different personal and environmental factors. Our study demonstrates that higher perceived levels self-efficacy, social support, and workload and autonomy are negatively associated with the duration of sick leave, consistent with previous studies (Amick et al., 2004; Lysaght and Larmour-Trode, 2008; Boštjančič and Koračin, 2014; Hoefsmit et al., 2014; Volker et al., 2015; Lork and Holmgren, 2018). The results further indicate that there is an association between the duration of sick leave and employees’ work satisfaction after returning to work. Our findings were mostly consistent with previous research (e.g., Amick et al., 2004; Dionne et al., 2007; Lysaght and Larmour-Trode, 2008; Boštjančič and Koračin, 2014; Hoefsmit et al., 2014), except as regards perceived workload (Haveraaen et al., 2016), where our hypothesis was not supported. Previous research indicates that a high perceived workload results in more prolonged sick leave (Haveraaen et al., 2016), but our results showed that the male participants perceived their workloads as higher following a shorter sick leave compared to a longer sick leave, which could be explained by those returning after a longer sick leave being exposed to less demands at work.

This study puts into the spotlight an extremely important topic for all employers and employees, with the number of the answers to the open-ended questions showing that returning to work is seen as very important by staff. The results also indicate that there is plenty room for improvement on how to help people who are returning to work, with social engagement confirmed as one of the most important factors for work satisfaction after returning to one’s job (Boštjančič and Koračin, 2014).

The results can have high value for all professionals dealing with employees who are returning to work (Boštjančič and Koračin, 2014; Hoefsmit et al., 2014), as they indicate which factors need to be considered so as to ease the process of returning to work and reduce the length of absences. In addition to this, the results can serve as the basis for creating return-to-work programs, especially for employees that are on long sick leaves. We agree with previous studies (Lysaght and Larmour-Trode, 2008; Haveraaen et al., 2016) that when helping employees on long-term sick leave, employers have to focus on factors such as self-efficacy (Volker et al., 2015), instead of on the symptoms of their conditions. Employees returning to work place high value on having flexible, empathetic, and supportive superiors.

However, in our opinion the study’s most important contribution is that it draws attention to a very important topic that is often overlooked or taken too lightly. Since both work and health are important for one’s wellbeing, this field deserves special attention.

One of the limitations of the present study is that the questionnaire consisted of four independent scales, therefore it was long and this meant that a high percentage of participants did not finish it, so we could not use their answers. Another potential limitation of the present study could be that there was a gender discrepancy in our sample, which usually happens in this type of research. Moreover, there was a bigger gender imbalance in the long-term sick leave group – due to the inclusion of maternity leave – than in the short-term group, although maternity leave is not considered a form of sick leave, but instead just an absence from work. The greater gender imbalance in the long-term sick leave group may also have affected the overall comparison between the long- and short-term sick leave groups, a further limitation of this work. An additional limitation of the study is that, when evaluating the factors involved in the duration of sick leave, we did not focus on the contribution of the condition that prompted the leave, which we believe could be the focus of further research. Another limitation could be that we did not compare the reasons for sick leave within the two independent test groups – shorter and longer sick leave – which could potentially affect the results as well. Furthermore, one of the limitations of our research is that the participants who were on longer sick leave had generally also been back at work for a longer period when responding to the questionnaire than those who were on shorter sick leave. We suggest these factors be studied in more detail in further research. Nonetheless, the aim of this study was to look more broadly, since many extant studies focus on specific target groups, there is still a lot of room for further investigation, especially within each of the variables we looked into and within the various reasons for being on sick leave in the first place.

## Conclusion

The present research indicated that returning to work is a very important topic which should be given more attention. Moreover, it is the first study focusing on this topic in Slovenia and could present a solid starting point for future research. The return-to-work process can be affected by personal, social, and occupational factors. In more detail, higher perceived levels self-efficacy, social support, and workload and autonomy are negatively associated with the duration of sick leave. These factors can influence the duration of sick leave as well as the employee’s overall work satisfaction after returning to work. The responsibility for a successful return-to-work process should not only be in the hands of employees, and thus employers should take into consideration the previously mentioned factors and thoughtfully include them when planning return-to-work programs.

## Data Availability Statement

The datasets generated for this study are available on request to the corresponding author.

## Ethics Statement

The administration adhered to the requirements of privacy in the Slovene Law, followed and approved by the Ethical Committee at the Faculty of Arts, University of Ljubljana. The consent of the participants was obtained by virtue of survey completion.

## Author Contributions

All authors listed have made a substantial, direct and intellectual contribution to the work, and approved it for publication.

## Conflict of Interest

The authors declare that the research was conducted in the absence of any commercial or financial relationships that could be construed as a potential conflict of interest.

## Footnotes

1 www.1ka.si
